# Study on Grinding-Affected Layer of Outer-Ring Inner Raceway of Tapered Roller Bearing

**DOI:** 10.3390/ma16227219

**Published:** 2023-11-17

**Authors:** Dameng Cheng, Guangdi Jin, Yufei Gao, Panling Huang, Zhenyu Shi, Yuanchao Tang

**Affiliations:** 1 Key Laboratory of High Efficiency and Clean Mechanical Manufacture of MOE, School of Mechanical Engineering, Shandong University, Jinan 250061, China; 2 Shandong Chaoyang Bearing Co., Ltd., Dezhou 253200, China

**Keywords:** tapered roller bearing, raceway, grinding-affected layer, grinding, temperature field

## Abstract

In the grinding of bearing raceways, the coupling effect between grinding force and heat in the contact area between the grinding wheel and the workpiece causes changes in the material structure and mechanical properties of the raceway surface layer, which can lead to the formation of a grinding-affected layer. The grinding-affected layer has a significant impact on the service performance and fatigue life of bearings. In order to improve the ground surface quality of the outer-ring inner raceway of tapered roller bearings and optimize the processing parameters, this paper presents a study on the grinding-affected layer. A finite element simulation model for grinding the outer-ring inner raceway of the tapered roller bearing was established. The grinding temperature field was simulated to predict the affected-layer thickness during raceway grinding. The correctness of the model was verified through grinding experiments using the current industrial process parameters of bearing raceway grinding. The research results indicate that the highest grinding temperature of the outer-ring inner raceway of the tapered roller bearing is located near the center of the grinding arc area on the thin end edge. As the workpiece speed and grinding depth decrease, the highest grinding temperature decreases, and the dark layer thickness of the grinding-affected layer decreases or even does not occur. The research results can provide theoretical guidance and experimental reference for grinding the raceway of tapered roller bearings.

## 1. Introduction

High-precision tapered roller bearings are widely used in aerospace, high-speed trains, precision machine tools, and other fields. They guarantee long-term high-precision, low vibration, and low noise operation of the industrial equipment [1,2,3]. The raceway surface of tapered roller bearings is the main working surface, which will bear the influence of high speed, high load, and high temperature during operation. And the raceway surface is also the part that is relatively easy to damage. The surface processing quality of the tapered roller bearing raceway directly determines the stability and service life of the bearing [4].

Grinding is an important process for machining the inner raceway surface of the bearing outer ring [5,6]. Grinding the outer ring of bearings is a complex process, involving complex machining mechanisms, a high-grinding-specific energy, and the coupling of multiple process variables. Therefore, it has been difficult to explain the characteristics of the machining process accurately and systematically [7,8,9]. Wakabayashi et al. [10] investigated the tribological action of minimal quantity lubrication (MQL) media and atmospheric carrier gases. The investigation demonstrated that their adsorption behavior onto metal surfaces was closely connected to the cutting performance of a synthetic polyol ester and carrier gases in practical MQL machining. The author conducted MQL machining on aluminum. The result showed that the presence of oxygen resulted in unfavorable cutting phenomena. In addition, when machining large-aspect-ratio and thin-walled parts, the grinding accuracy is difficult to ensure. Onishi et al. [11] developed a new method for grinding slender shafts without an auxiliary device. Transverse grinding tests were carried out on the workpiece with different wheel speeds. When the grinding wheel speed is higher, the normal grinding force is smaller and the shape accuracy of the grinding workpiece is improved. And at higher grinding wheel speeds, the normal grinding force can be kept low even if the transverse speed is set higher. Sun et al. [12] came to the same conclusion that increasing the wheel line speed is an effective way to keep the grinding force at a low level and achieve high efficiency. The complexity of grinding can be seen from the above research content. The grinding quality is not only affected by the machining parameters, but also by the machining medium, the vibration of the machine tool, and the shape of the workpiece. Ullah et al. [13] studied the grinding mechanism through theory and experiment. A grinding mechanism model considering the size, height, and uneven distribution of abrasive particles was established to predict the surface topography of the workpiece. Although the theoretical grinding mechanism model does not incorporate the thermal/elastic/plastic deformations and machine/grinding wheel stiffness, the model calculation results are in good agreement with the experiment. The results show that the loss/truncation/wear of abrasive grains, the loss/truncation/wear of abrasive grains, and the roughness of the already-ground workpiece surface are the main factors that govern the grinding mechanism.

During the grinding, a grinding-affected layer will be formed on the surface of the bearing raceway that is different from the material bulk structure. The outermost layer of the grinding-affected layer is called the white layer because it appears white under an optical microscope [14]. The subsurface layer of the grinding-affected layer is called the dark layer because it appears black under an optical microscope [15]. The white layer has high hardness but high brittleness, which can easily lead to cracks on the raceway surface. The dark layer is mainly composed of tempered martensite, tempered sorbite, tempered troostite, and other tempered structures. The generation of the dark layer will reduce the hardness of the surface and eventually reduce the fatigue strength of the raceway. Therefore, the appearance of the grinding-affected layer should be avoided in the grinding.

To deeply understand the grinding-affected layer and reveal the formation and evolution mechanism of the grinding-affected layer, scholars have carried out a lot of research on the grinding-affected layer during grinding. Huang et al. [16] conducted grinding experiments on hardened AISI52100 steel and studied the formation mechanism of the white layer. The results showed that the white layer was formed through the phase transition caused by grinding heat and rapid cooling. And it was found that the depth of the white layer increased with the increase in the grinding wheel speed. Gong et al. [17] studied the effect of grinding parameters on the characteristics of the grinding-affected layer. With the increase in grinding wheel line speed, the thickness of the grinding-affected layer decreases first and then increases. With the increase in grinding depth, the thickness of the grinding-affected layer increases continuously. With the increase in the workpiece feed speed, the thickness of the grinding-affected layer increases first and then decreases. Chang et al. [18] studied the influence of grinding on the grinding-affected layer of the bearing groove through a full factor experiment. The results indicated that the rotational speed of the grinding wheel was the main factor affecting the grinding-affected layer. When the rotational speed of the grinding wheel increases, the thickness of the dark layer thickness increases gradually. Mao et al. [19] observed and measured the grinding-affected layer structure through an optical microscope. It was found that as the grinding depth increased, the thickness of both the white and dark layers increased. The dark layer thickness was greater than the white layer thickness.

On the other hand, grinding has a high specific energy, that is, the energy required to remove a unit volume of material is large. Most of the energy is converted into heat energy. A part of the heat energy is dispersed in the air and coolant. And a considerable part of the heat energy is transmitted to the workpiece, resulting in local high temperature in the grinding area, which affects the grinding surface quality and performance. Many studies have shown that grinding temperature has a direct effect on the formation and evolution of the grinding-affected layer. Stead [20] proposed a model for the formation mechanism of the white layer. The results indicated that when the temperature in the grinding area reached the α-γ phase transformation temperature, the surface material underwent austenitization. The temperature then rapidly decreased and the martensitic structure formed through quenching. Mao et al. [21] conducted grinding experiments on GCr15 bearing steel and observed the characteristics of the white layer. The results indicated that the white layer also appeared when the grinding temperature was lower than the phase transformation temperature. The thickness of the white layer suddenly increased when the temperature exceeded the phase transformation temperature. Jiang et al. [22] analyzed the effect of grinding parameters on the white layer. As the grinding depth and speed increased, the grinding temperature increased. And the combination of high grinding temperature and mechanical extrusion caused delamination of the white layer.

Exploring the influence of temperature on the grinding-affected layer requires accurate measurement of the real-time temperature during the grinding. There are currently two main methods for temperature measurement, namely thermal imaging temperature measurement and thermocouple temperature measurement. For the thermal imaging temperature measurement method, in the case of wet grinding, the grinding coolant will cause significant interference for the experimental results [23]. For the thermocouple temperature measurement method, it is suitable for the flat surface grinding and is rarely used for grinding the inner raceway of the bearing outer ring. Based on this, scholars have studied the grinding temperature field through simulation analysis methods and have predicted the grinding-affected layer based on the temperature field. Zhao [24] studied the effect of grinding parameters on grinding temperature and the formation mechanism of the grinding-affected layer through molecular dynamics. The results showed that the high grinding temperature caused the phase transformation of the surface material to form the grinding-affected layer. Zhang et al. [25] simulated and analyzed the temperature field distribution of the workpiece under grinding hardening conditions using the finite element method. The depth of the hardened layer was derived based on the temperature distribution and martensitic transformation conditions. Guo et al. [26] combined the finite element and Cellular Automation (CA) methods to study the distribution and variation of the grinding temperature field. The transformation process of ‘austenite-martensite’ was simulated, and the hardening depth under different grinding parameters was predicted. Mahdi et al. [27] conducted the two-dimensional finite element simulation of the grinding temperature field and predicted the depth of martensitic transformation of alloy steel. Du [28] conducted simulation analysis on the deep groove ball bearing groove grinding. The influence of processing parameters on the depth of the grinding-affected layer was investigated. The results showed that the increase in grinding depth and wheel speed would increase the depth of the grinding-affected layer.

In summary, the grinding-affected layer on the grinding surface has a great impact on the performance and service life of the bearing. The appearance of the grinding-affected layer should be avoided in the grinding. Grinding temperature has an important effect on the formation and evolution of the grinding-affected layer. The formation and depth of the grinding-affected layer can be predicted by analyzing the grinding temperature field. However, the real-time measurement error of the temperature field during grinding is large, so the finite element simulation is a common method to analyze the temperature field. At present, relevant scholars have carried out extensive research on the grinding-affected layer of the bearing raceway. However, most of the research content involves the flat surface grinding of the bearing materials. And there is no relevant report on the grinding of the outer-ring inner raceway surface of the tapered roller bearing.

Therefore, taking the TR0708 tapered roller bearing outer ring as the research object, the variation in the dark layer thickness was analyzed during the grinding of the inner raceway in this paper. Firstly, a heat flux density model was established based on the different interaction stages between abrasives and the workpiece. The heat flux density model was imported into ABAQUS (finite element analysis software https://www.think-s.com/) for temperature field simulation. According to the simulation results, the grinding-affected layer characteristics were analyzed. And the correctness of the finite element model was verified based on the grinding experimental results. The research results provide a theoretical basis and experimental reference for improving the grinding surface quality of the outer-ring inner raceway of the tapered roller bearing and eliminating the grinding-affected layer. It has important guiding significance for optimizing process parameters and improving grinding efficiency.

## 2. Model of Grinding Force and Heat Flux Density

### 2.1. Grinding Contact Arc Length and Maximum Undeformed Thickness

The research results of Guo [29] and Mao [30] indicated that most of the grinding heat was transferred to the workpiece in the grinding arc zone. Therefore, the grinding contact arc length is an important parameter for calculating the heat flux density, which can be expressed as

(1) $$l_{c}=\sqrt{a_{e}d_{\mathrm{se}}}$$

where *d*_se_ is the diameter of the grinding wheel; *a*_e_ is the grinding depth, *a*_e_ = 1/2*v*_f_(60/*n*_w_); *v*_f_ is the feed rate of the grinding wheel; and *n*_w_ is workpiece rotational speed.

The cutting depth *h*_cuz_ of abrasives is the difference between the protrusion height *h* and the minimum protrusion height *h*_min_ of abrasives. The maximum undeformed thickness *h* is the maximum cutting depth generated by the maximum protrusion height *h*_max_ of abrasives. The maximum undeformed thickness is an important parameter in solving the grinding force, which is related to abrasive density, the grinding wheel speed, the workpiece speed, and the grinding depth. It can be expressed as [31]

(2) $$h_{cu,\max}={\left(\frac{4}{N_{s}r}\left(\frac{v_{w}}{v_{s}}\right){\left(\frac{a_{e}}{d_{se}}\right)}^{\frac{1}{2}}\right)}^{\frac{1}{2}}$$

where *N*_s_ is the surface abrasive density; *r* is the cutting thickness ratio, where *r* = 10; *v*_w_ is the workpiece speed; and *v*_s_ is the grinding wheel speed.

### 2.2. Calculation of the Number of Abrasives

When the abrasives cut into the workpiece very shallowly, the interaction between the abrasives and the workpiece is the plowing stage. Therefore, when calculating the grinding force, the sliding stage can be ignored. The number of abrasives in the plowing and cutting stages only needs to be calculated. Firstly, *x* and *y* are defined to describe the size and protrusion height of abrasives, respectively. And variable *l* is defined to describe the position of abrasives. Based on the size, protrusion height, and position of abrasives at different stages, the definition domains of *x*, *y*, and *l* are obtained. Then, the number of abrasives at different stages can be calculated through integration. The probability density functions *P*_g_(*x*), *P*_g_(*y*), and *P*_g_(*l*) of *x*, *y*, and *l*, respectively, can be expressed as [23].

(3) $$\left\{\begin{array}{l} P_{g}\left(x\right)=\frac{6}{\delta \sqrt{2\pi }}e^{-\frac{1}{2}{\left(\frac{6}{\delta }x\right)}^{2}}dx\\ P_{g}\left(y\right)=\frac{1}{h_{cu,\max}}dy\\ P_{g}\left(l\right)=\frac{1}{l_{c}}dl \end{array}\right.$$

The domain is defined as

(4) $$\left\{\begin{array}{l} x\in \left[-\delta /2,\delta /2\right]\\ y\in \left[d_{\max}-h_{\mathrm{cu},\max},d_{\max}\right]\\ l\in \left[0,l_{c}\right] \end{array}\right.$$

where *δ* is the difference between the largest and smallest abrasive size.

The stage of action between the abrasives and the workpiece is determined by the size and the cutting depth of the abrasives. The equations of the critical depth *h*_plow_ of a single abrasive from the sliding stage to the plowing stage and the critical depth *h*_cut_ of a single abrasive from the plowing stage to the cutting stage, respectively, can be expressed as

(5) $$\left\{\begin{array}{l} h_{\mathrm{plow}}=\xi_{\mathrm{plow}}d_{\mathrm{gx}}\\ h_{\mathrm{cut}}=\xi_{\mathrm{cut}}d_{\mathrm{gx}} \end{array}\right.$$

where the coefficient *ξ*_plow_ ≈ 0.015 and *ξ*_cut_ ≈ 0.025 [32].

According to the critical depth, the equation *l*_slid_, *l*_plow_, and *l*_cut_ for the position of the abrasives in the sliding stage, plowing stage, and cutting stage, respectively, can be expressed as [23]

(6) $$\left\{\begin{array}{l} l_{\mathrm{slid}}=l_{c}\frac{d_{\max}-y}{h_{\mathrm{cu},\max}}\\ l_{\mathrm{plow}}=l_{c}\frac{\xi_{\mathrm{plow}}d_{\mathrm{gx}}+d_{\max}-y}{h_{\mathrm{cu},\max}}\\ l_{\mathrm{cut}}=l_{c}\frac{\xi_{\mathrm{cut}}d_{\mathrm{gx}}+d_{\max}-y}{h_{\mathrm{cu},\max}} \end{array}\right.$$

When *l*_plow_ > *l*_c_, the abrasives are in the non-contact and sliding stage. When *l*_cut_ > *l*_c_ > *l*_plow_, the abrasives are in the non-contact, sliding, and plowing stages. When *l*_c_ > *l*_cut_, the abrasives are in the non-contact, sliding, plowing, and cutting stages. Using *l*_plow_ = *l*_c_ and *l*_cut_ = *l*_c_, the following two straight line equations *f*(*x*) and *g*(*x*), respectively, can be expressed as

(7) $$\left\{\begin{array}{l} y=f\left(x\right)=\xi_{\mathrm{plow}}\left(x+d_{\mathrm{mean}}\right)+d_{\max}-h_{\mathrm{cu},\max}\\ y=g\left(x\right)=\xi_{\mathrm{cut}}\left(x+d_{\mathrm{mean}}\right)+d_{\max}-h_{\mathrm{cu},\max} \end{array}\right.$$

According to *ξ*_plow_ ≈ 0.015 and *ξ*_cut_ ≈ 0.025, it can be known that the two lines *y* = *f*(*x*) and *y* = *g*(*x*) are not parallel and the intersection point is *x* = −*d*_mean_. The two lines divide the XY plane into three regions: *I* < *f*(*x*), *f*(*x*) < *II* < *g*(*x*), and *III* > *g*(*x*).

From the above description, region *I* contains the non-contact and sliding abrasives; region *II* contains non-contact, sliding, and plowing abrasives; and region *III* contains non-contact, sliding, plowing, and cutting abrasives. Inputting *y* into *g*(*x*), the following is obtained:

(8) $$x_{p}=\frac{1}{\xi_{\mathrm{cut}}}h_{\mathrm{cu},\max}-d_{\mathrm{mean}}$$

The probability equation of the number of plowing and cutting abrasives can be expressed as

(9) $$P_{*}=\int_{x_{\min}}^{x_{\max}}\int_{y_{\min}}^{y_{\max}}\int_{l_{\min}}^{l_{\max}}P_{g}(x)P_{g}(y)P_{g}(l)dldydx$$

When *x*_p_ > *δ*/2, *h*_cu,max_ > *d*_max_*ξ*_cut_. When *x*_p_ < *δ*/2, *h*_cu,max_ < *d*_max_*ξ*_cut_. By judging the conditions of the *h*_cu,max_ value, the integral interval in Formula (9) is determined. The integration interval is shown in Table 1.

After calculating the probability of the number of plowing and cutting abrasives using Equation (9), the equation for the number of plowing and cutting abrasives in the grinding arc area can be expressed as [23]

(10) $$N_{*}=l_{c}b_{w}h_{\mathrm{cu},\max}N_{v}P_{*}$$

where the symbol ‘∗’ in ‘*P*_∗_’ and ‘*N*_∗_’ is ‘plow or cut’, indicating plowing and cutting abrasives, respectively.

### 2.3. Grinding Force of Single Abrasive Grain

Hecker used the definition and derivation method of Brinell hardness to inversely derive the grinding force of a single plow abrasive, and the equation is

(11) $$H=\frac{2F}{\pi D\left(D-\sqrt{D^{2}-b^{2}}\right)}$$

where *F* is the pressure, *D* is the diameter of the cemented carbide ball, *b* is the indentation diameter, and $\left(D-\sqrt{D^{2}-b^{2}}\right)/2$ is the cutting depth *h*_cuz_ of the abrasives.

**Figure 1:**
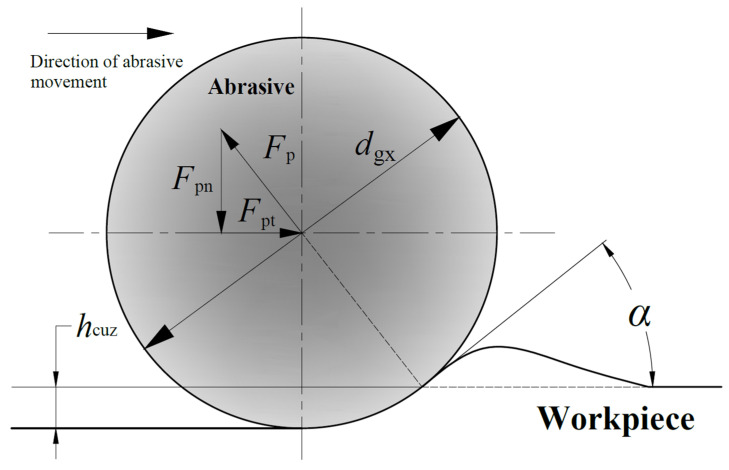
Force analysis diagram of a single abrasive plowing workpiece.

The force analysis of a single abrasive plowing workpiece is shown in Figure 1. *F*_p_ is the grinding force of the plowing abrasive. *F*_pn_ is the normal force of the plowing abrasive. *F*_pt_ is the tangential force of the plowing abrasive. *d*_gx_ is the diameter of the abrasive. *α* is the cutting angle.

Hecker replaced the diameter *D* of the hard alloy ball in Equation (11) with the abrasive diameter *d*_gx_ and derived the plowing force *F*_p_ of a single abrasive [33]:

(12) $$F_{p}=\pi Hd_{gx}h_{\mathrm{cuz}}$$

Chen et al. [34] further derived the equations for plowing normal force *F*_pn_ and plowing tangential force *F*_pt_, respectively:

(13) $$\left\{\begin{array}{l} F_{\mathrm{pt}}=\pi Hd_{\mathrm{gx}}h_{\mathrm{cuz}}\left(\sin\alpha +\mu_{d}\cos\alpha \right)\\ F_{\mathrm{pn}}=\pi Hd_{\mathrm{gx}}h_{\mathrm{cuz}}\left(\cos\alpha -\mu_{d}\sin\alpha \right) \end{array}\right.$$

where *μ*_d_ is the friction coefficient between the workpiece and the abrasives.

The cutting angle *α* can be expressed as

(14) $$\alpha =\arccos\left(1-\frac{2h_{\mathrm{cuz}}}{d_{\mathrm{gx}}}\right)$$

**Figure 2:**
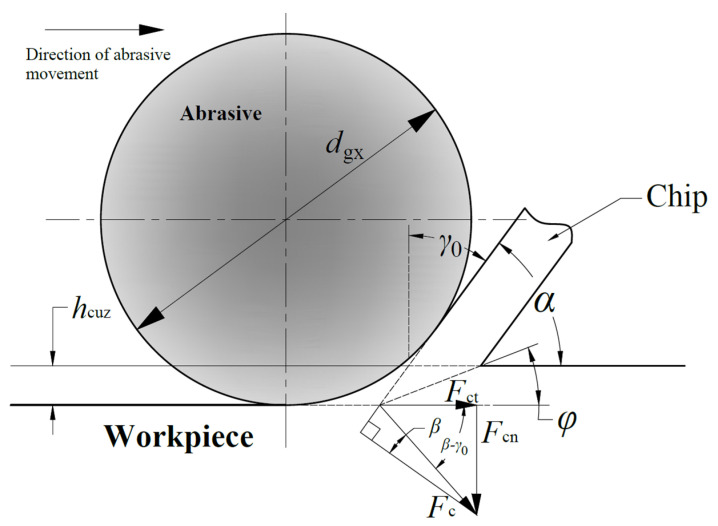
Force analysis diagram of a single abrasive cutting workpiece.

Equations (12) and (13) only apply to the plowing stage. For the abrasives in the cutting stage, a new cutting force model needs to be established. The force analysis of a single abrasive cutting workpiece is shown in Figure 2. *F*_c_ is the grinding force of the cutting abrasive. *F*_cn_ is the normal force of the cutting abrasive. *F*_ct_ is the tangential force of the cutting abrasive. *γ*_0_ is the front corner. *φ* is the shear angle. *β* is the friction angle.

Jiang et al. established expressions of the single abrasive grinding force in the cutting stage [23]:

(15) $$\begin{array}{l} \left\{\begin{array}{l} F_{\mathrm{ct}}=\tau_{s}\frac{h_{\mathrm{cuz}}d_{\mathrm{gx}}}{4}{\left(\frac{\cos\gamma_{0}}{\sin\varphi \cos\left(\varphi -\gamma_{0}\right)}\right)}^{n}\frac{\cos\left(\beta -\gamma_{0}\right)}{\sin\varphi \cos\left(\varphi +\beta -\gamma_{0}\right)}\\ F_{\mathrm{cn}}=\pi \tau_{s}\frac{h_{\mathrm{cuz}}d_{\mathrm{gx}}}{4}{\left(\frac{\cos\gamma_{0}}{\sin\varphi \cos\left(\varphi -\gamma_{0}\right)}\right)}^{n}\frac{\sin\left(\beta -\gamma_{0}\right)}{\sin\varphi \cos\left(\varphi +\beta -\gamma_{0}\right)} \end{array}\right. \end{array}$$

where *τ*_s_ is the shear yield strength of the material. *n* is the strengthening coefficient, and for GCr15 bearing steel, *n* = 0.15.

The front angle *γ*_0_, the shear angle *φ,* and the friction angle *β* can be expressed as

(16) $$\gamma_{0}=-\arcsin\left(1-\frac{2h_{\mathrm{cuz}}}{d_{\mathrm{gx}}}\right)$$

(17) $$\varphi =\frac{\pi }{4}-\frac{\beta }{2}+\frac{\gamma_{0}}{2}$$

(18) $$\beta =\arctan\mu_{g}$$

where *μ*_g_ is the friction coefficient between the grinding chips and the abrasives.

According to the above equations, the bearing raceway is discretized; the inner diameter, the linear speed, and the grinding force of each bearing raceway section are calculated; and then the grinding force of each bearing raceway section is calculated. The grinding force during grinding of the tapered roller bearing raceway can be obtained by summing up each result.

### 2.4. Grinding Heat Flux Density

According to the tangential component force *F*_tl_ of the grinding force, the total heat source *q*_l_ in the grinding arc can be expressed as

(19) $$q_{l}=F_{\mathrm{tl}}v_{s}/\left(b_{w}l_{c}\right)$$

The heat distribution ratio between the grinding wheel and the workpiece is [35]

(20) $$\varepsilon_{\mathrm{ws}}=\frac{1}{1+\frac{0.97k_{g}}{\sqrt{r_{0}v_{s}{\left(k\rho c\right)}_{w}}}}$$

where *k*_g_ is the thermal conductivity coefficient of the abrasive, and *k*_w_, *ρ*_w_, and *c*_w_ are the thermal conduction coefficient, density, and specific heat capacity of the workpiece material, respectively. And *r*_0_ is the contact radius of the abrasive. Rowe et al. [36] observed the abrasives on the surface of an alumina grinding wheel and calculated the heat distribution ratio with *r*_0_ = 15 μm.

The thermal diffusion coefficient of the workpiece *α*_w_ can be expressed as [23]

(21) $$\alpha_{w}=\frac{k_{w}}{\rho_{w}c_{w}}$$

The Peclet value reflects the degree to which the simulation temperature field is affected by the contact angle. The smaller the Peclet value is, the smaller the impact of the contact angle is [37]. The equation is as follows:

(22) $$Pe=\frac{v_{w}l_{c}}{4\alpha_{w}}$$

The heat flux density transmitted to the workpiece is

(23) $$q_{\mathrm{wl}}=\frac{\varepsilon_{\mathrm{ws}}\left(q_{l}-q_{\mathrm{chl}}\right)}{1+\varepsilon_{\mathrm{ws}}h_{f}/h_{w}}$$

where *q*_chl_ is the heat transferred into the chips. According to the motion trajectory of the abrasives, there are fewer chips during the grinding, so the heat transferred into the chips is not considered, *q*_chl_ = 0. *h*_f_ is the convective heat transfer coefficient of the grinding coolant. *h*_w_ is the heat transfer coefficient of the workpiece, and the equation is [23]

(24) $$h_{w}=\frac{3}{2C}\sqrt{\frac{k_{w}\rho_{w}c_{w}v_{w}}{l_{c}}}$$

where parameter *C* has the following equation relationship with the *Pe* value:

(25) $$C=\left\{\begin{array}{ll} 1.07 & Pe>10\\ \frac{0.95}{\pi }\sqrt{2\pi +Pe/2} & \;0.2<Pe<10\\ 0.76 & Pe<0.2 \end{array}\right.$$

The material properties are shown in Table 2. Inputting the material properties and grinding parameters into the above equations, the heat flux density transmitted into the workpiece can be obtained.

## 3. Finite Element Model of Grinding Temperature Field

### 3.1. Simulation Analysis Process

**Figure 3:**
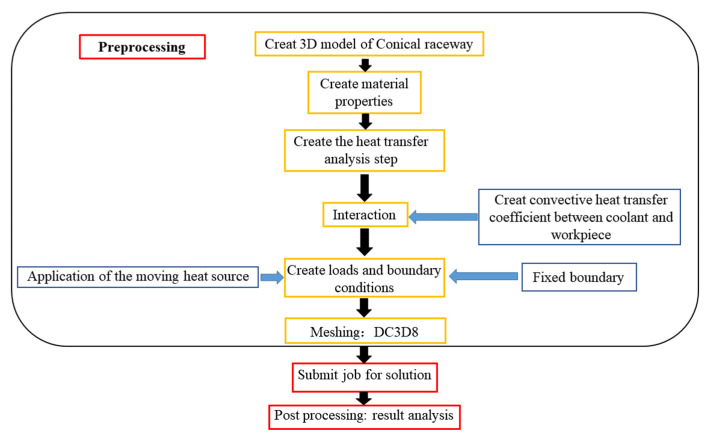
Simulation flow chart.

The heat flux density model in Section 2 was input into the finite element model to simulate the grinding of the outer-ring inner raceway of the tapered roller bearing, and the transient temperature field distribution was calculated. This paper is based on ABAQUS software (https://www.think-s.com/) for finite element analysis, and the specific simulation analysis process is shown in Figure 3. First, a three-dimensional model of the outer ring of tapered roller bearings was established, and the material properties of GCr15 were assigned. Then, a heat transfer analysis step was created to calculate the temperature field, and the interaction properties, boundary conditions, and heat source loads corresponding to the actual processing were applied. The post-processing stage involves statistical analysis of the calculation results.

The outer ring of the TR0708 tapered roller bearing is made of hardened bearing steel GCr15. Its thermal physical properties vary with temperature. And the thermal physical properties in Table 3 were inputted into the finite element model.

### 3.2. Finite Element Model

**Figure 4:**
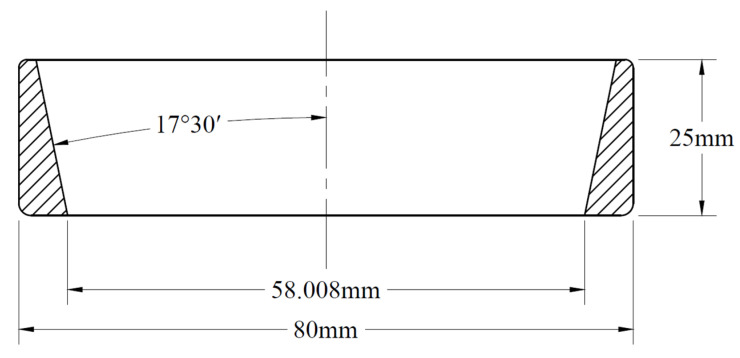
Cross-sectional view of the outer ring of TR0708 tapered roller bearing.

**Figure 5:**
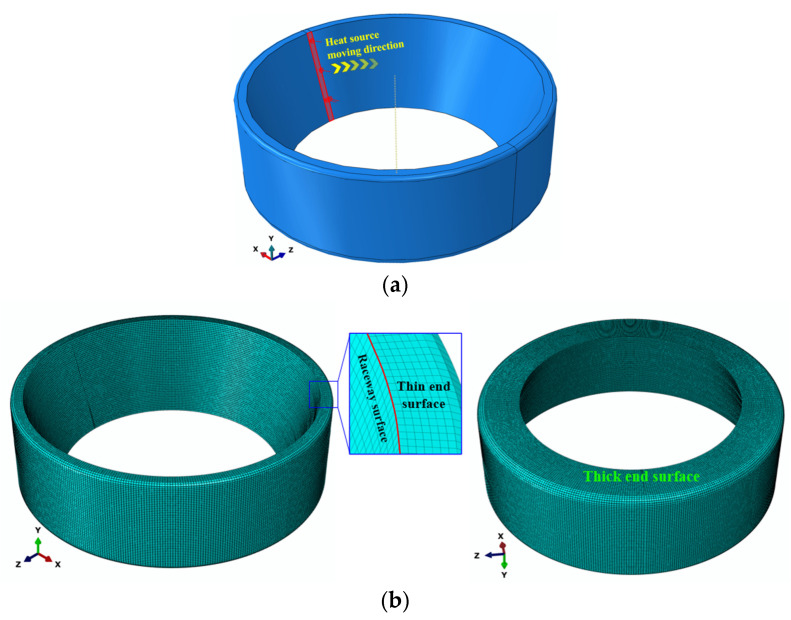
Finite element model of the outer ring of tr0708 tapered roller bearing, (**a**) Schematic diagram of three-dimensional heat source movement, (**b**) meshing.

The outer ring of the TR0708 tapered roller bearing was taken as the research object. The finite element model was established according to its actual size. The geometric dimensions of the bearing outer ring are shown in Figure 4, and the inner diameter of the outer ring × external diameter × height × angle was *Φ*58.008 mm × *Φ*80 mm × 25 mm × 17°30′. The three-dimensional model and meshing of the outer ring are shown in Figure 5. The heat source distribution model is directly related to the calculation accuracy. In this paper, a triangular heat source distribution model was established in the finite element model [38]. The moving heat source was applied onto the inner raceway surface of the outer ring, as shown in Figure 5a. It can be updated continuously along the grinding direction to realize the moving heat source applied on the model and to simulate the grinding temperature field. The surface convection heat transfer coefficient of the outer-ring inner raceway generated by the coolant was 15,000 W/m^2^K. The initial temperature and ambient temperature were set to 20 °C. In finite element analysis, the element size and density will affect the simulation results and computational efficiency. Considering the calculation time cost and calculation accuracy comprehensively, the elements size was set to 1 mm, as shown in Figure 5b, and the mesh independence was verified.

## 4. Experimental Equipment and Methods

**Figure 6:**
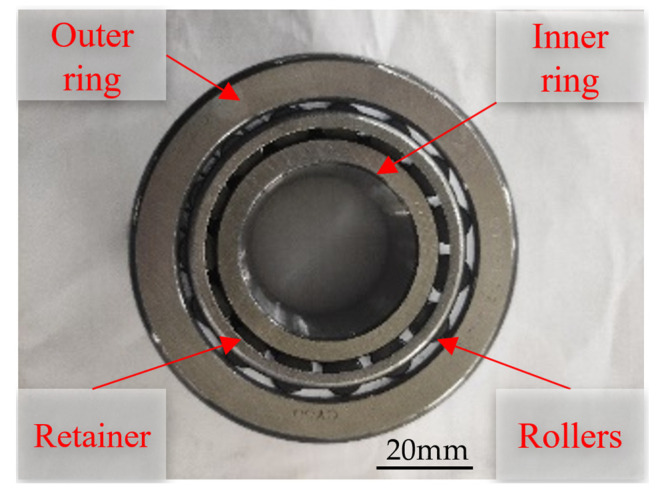
TR0708 tapered roller bearing.

The TR0708 tapered roller bearing selected for the experiment is shown in Figure 6. Grinding experiments were conducted on the inner raceway of the bearing outer ring.

**Figure 7:**
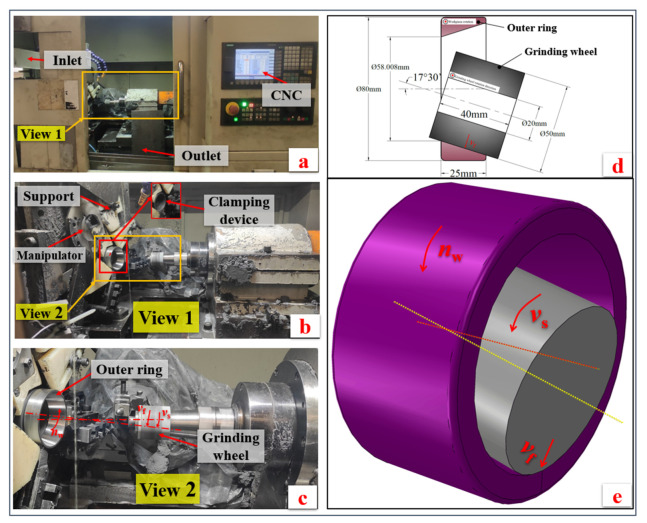
(**a**) CNC grinding machine, (**b**) enlarged view of processing region, (**c**) relative motion diagram of grinding, (**d**) schematic diagram of relative position between grinding wheel and outer ring, (**e**) 3D schematic diagram of grinding.

The CNC grinding machine produced by Pusen Precision Machine Tool Manufacturing Co., Ltd., Wuxi, China, was used in the grinding experiment, as shown in Figure 7a. Figure 7b shows an enlarged view of the processing area. Figure 7c shows the motion direction diagram of the grinding. Figure 7d is the relative position diagram of the grinding wheel and the outer ring, and Figure 7e shows the 3D schematic diagram of the grinding. Automatic loading and unloading by the manipulator and magnetic clamping device are adopted. The grinding wheel is made of white corundum with a ceramic bond. The grain size is 100#, the outer diameter is 50 mm, the aperture is 20 mm, the thickness is 40 mm, and the organization number is 7. The experiment adopts inner circle clockwise wet grinding, and the type of grinding fluid is water-soluble emulsion.

To explore the influence of machining parameters on the grinding-affected layer and to achieve the balance between surface quality and machining efficiency, it is necessary to choose a larger grinding wheel linear speed. Therefore, *v*_s_ = 40 m·s^−1^ was selected to carry out grinding experiments. The designed experimental scheme and processing parameters are shown in Table 4.

**Figure 8:**
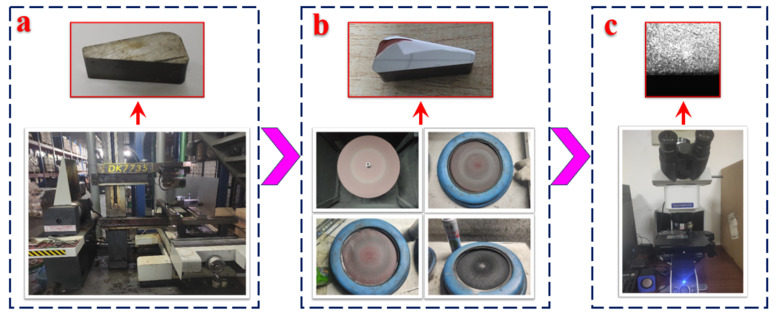
Detection of grinding-affected layer, (**a**) wire-cutting bearing outer raceway, (**b**) polishing, (**c**) microscopic observation.

After grinding, a sample with 20 mm of thickness was cut from the bearing outer ring using the wire-cutting equipment shown in Figure 8a. The 200#, 600#, and 2000# metallographic sandpapers and polishing cloth were used in sequence to polish the cross-section of the bearing outer ring sample until it appeared specular, as shown in Figure 8b. After cleaning the sample, the cross-section of the sample was corroded with 4% nitrate alcohol solution and then quickly rinsed with distilled water. After the sample processing was completed, the microstructure of the cross-section was observed under an optical microscope, as shown in Figure 8c.

## 5. Results and Discussion

### 5.1. Validation of Finite Element Models

For GCr15 hardened bearing steel, when the grinding temperature exceeds 150 °C, the martensite is tempered to form the dark layer. Based on this premise, the thickness of the dark layer can be determined according to the simulation results of the grinding temperature field.

**Figure 9:**
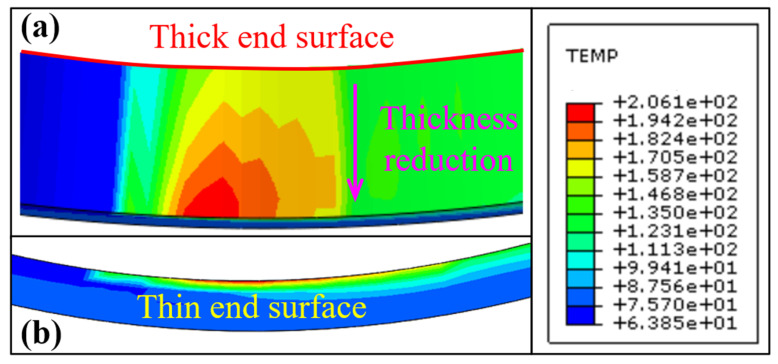
Cloud diagram of temperature field distribution on the outer ring raceway, (**a**) outer ring inner raceway surface, (**b**) Outer ring thin end surface.

Figure 9 shows the distribution of the simulated temperature field corresponding to the experimental parameters of Group 5. Figure 9a shows the grinding temperature field distribution on the outer-ring inner raceway surface. Figure 9b shows the temperature field cloud diagram distribution in the thickness direction of the thin end surface. The temperature field distribution has the following characteristics: The closer the grinding arc zone is, the higher the temperature, and the temperature decreases along the grinding direction. Along the width direction of the outer-ring inner raceway, the thicker the raceway, the lower the temperature. The highest temperature is located near the center of the grinding arc zone on the thin end surface. The temperature at the thin end decreases from the inner surface to the outer surface in Figure 9b. The thickness of the dark layer was determined to be 30.6 μm by measuring the node coordinates of 150 °C and the node coordinates of the highest temperature in the thin end surface. Similarly, the thickness of the dark layer corresponding to each experimental group in Table 4 can be inferred according to the simulation results of the temperature field, as shown in Table 5.

**Figure 10:**
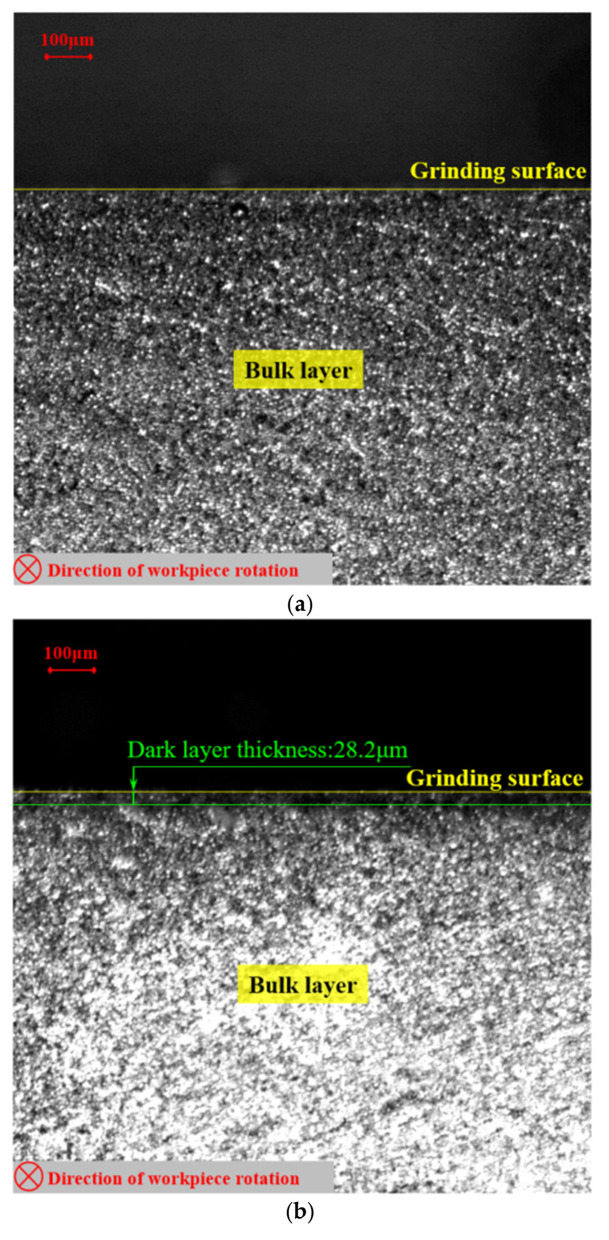

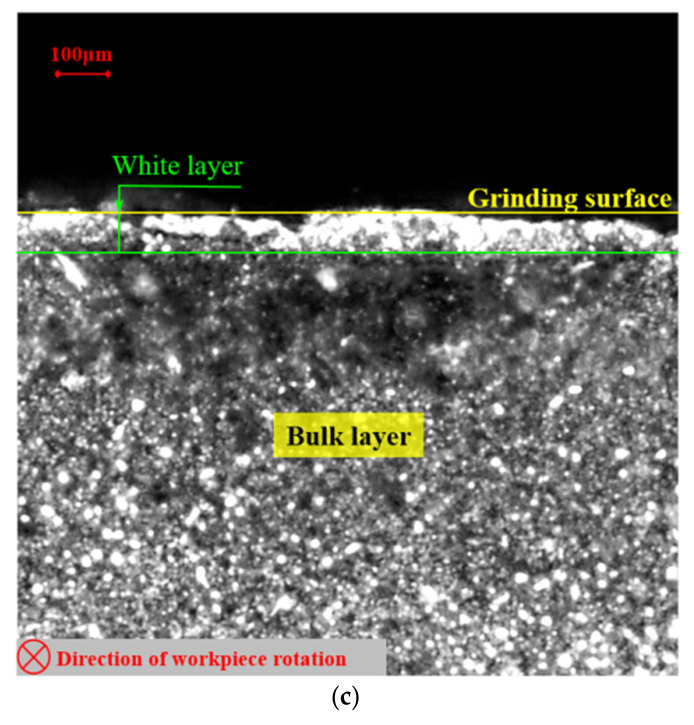
Microscopic photos of three sets of grinding experiments: (**a**) Group 1 (*v*_s_ = 40 m·s^−1^, *a*_e_ = 0.9 μm, *n*_w_ = 100 rpm), (**b**) Group 4 (*v*_s_ = 40 m·s^−1^, *a*_e_ = 1.5 μm, *n*_w_ = 100 rpm), (**c**) Group 8 (*v*_s_ = 40 m·s^−1^, *a*_e_ = 2.1 μm, *n*_w_ = 200 rpm).

The grinding-affected layer results of the grinding experiment are shown in Figure 10. Due to the different degree of corrosion in the preparation process of the sample, the microstructure observed under the microscope was different, but under specific processing parameters, the bulk structure, dark layer, and white layer can be obviously observed. Figure 10a shows the observation results of the sample in the Group 1 experiments. Only a uniform and continuous bulk structure was observed, and no grinding-affected layer was observed. This means that at lower workpiece speeds and grinding depths, the temperature in the grinding arc zone was small and did not reach the tempering temperature for the material. Figure 8b shows the observation results of the Group 4 experiments, where a dark layer with uniform thickness was observed. This is because the bulk structure undergoes instantaneous high-temperature tempering during grinding, forming tempered martensite, tempered sorbite, tempered troostite, and other tempered structures. But the grinding temperature did not reach the material phase transition temperature, and the austenitic structure in the subsurface will not transform into the martensite structure [19]. Within the observed range, three dark layer thickness values were taken from left to right intervals, and the average value was calculated to be 28.2 μm. In addition, discontinuous white layers were also observed in some experimental samples, as shown in the experimental results of Group 8 in Figure 10c. This is due to the random distribution of abrasives, which results in different instantaneous high temperatures and strains caused by a single grinding force, resulting in the appearance of white layers and significant thickness fluctuations [23].

**Figure 11:**
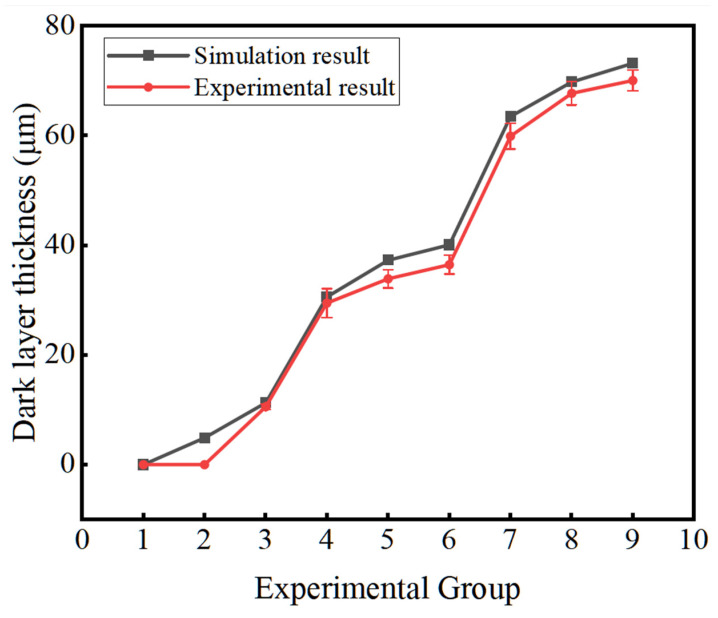
Comparison between experimental and simulation results of dark layer thickness.

The experimental and simulated results of the dark layer thickness were compared, as shown in Figure 11. The variation trend of the experimental and simulated results is the same, but the simulated results are slightly larger than the experimental results. The maximum difference is 5.4 μm, and the relative error is 9.8%, which is within the acceptable range, proving that the finite element model established in this paper for grinding the outer-ring inner raceway of tapered roller bearings is effective. The reasons for the errors may be due to the deviation between the finite element model, heat source distribution model, heat transfer model, meshing, and material parameters from actual conditions.

### 5.2. The Influence of Processing Parameters on the Temperature Field and Dark Layer Thickness during Grinding of the Outer-Ring Inner Raceway of the Tapered Roller Bearing

**Figure 12:**
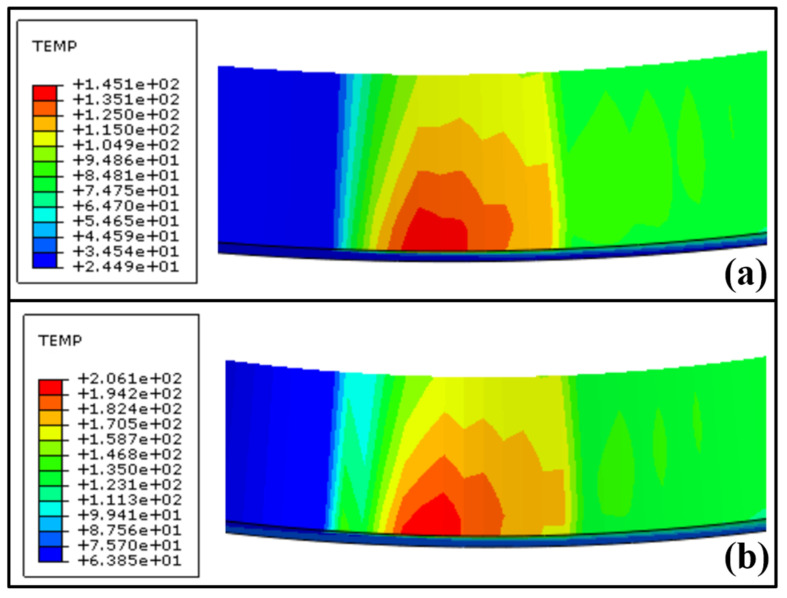
Cloud diagram of temperature field distribution with different grinding depths (*v*_s_ = 40 m·s^−1^, *n*_w_ = 100 rpm): (**a**) *a*_e_ = 0.9 μm, (**b**) *a*_e_ = 1.5μm.

Firstly, the influence of grinding depth on the grinding temperature field and dark layer thickness was analyzed. Figure 12 shows the finite element simulation results of the temperature field corresponding to two different grinding depths (*a*_e_ = 0.9 μm and *a*_e_ = 1.5 μm) when the grinding wheel line speed *v*_s_ = 40 m·s^−1^ and workpiece speed *n*_w_ = 100 rpm. When the grinding depth increased from 0.9 μm to 1.5 μm, the maximum temperature increased from 145.1 °C to 206.1 °C. With the increase in grinding depth, the maximum undeformed thickness increased, the penetration depth of abrasives increased, the number of abrasives involved in the plowing and cutting process increased, the normal grinding force increased, and the temperature in the grinding arc area rose rapidly.

**Figure 13:**
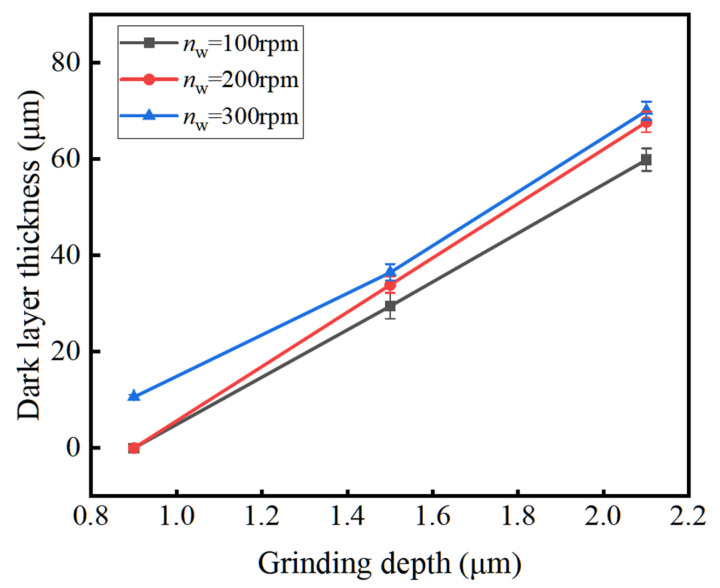
The influence of grinding depth on the dark layer thickness.

Figure 13 shows the influence of grinding depth on the dark layer thickness. With the increase in grinding depth, the dark layer thickness increases linearly. This is because with the increase in grinding depth, the maximum undeformed thickness increases, the normal grinding force increases, the temperature in the grinding arc area increases rapidly, and the dark layer thickness increases rapidly. The influence of grinding depth on the dark layer thickness is consistent with that of the temperature field.

**Figure 14:**
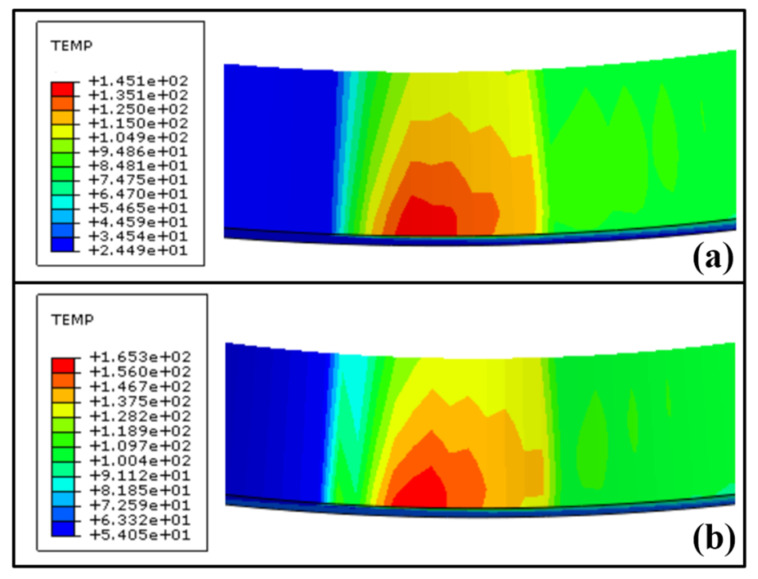
Cloud diagram of temperature field distribution with different workpiece speeds (*v*_s_ = 40 m·s^−1^, *a*_e_ = 0.9 μm): (**a**) *n*_w_ = 100 rpm, (**b**) *n*_w_ = 200 rpm.

Finally, the influence of workpiece speed on the grinding temperature field and dark layer thickness was analyzed. Figure 14 shows the finite element simulation results of the temperature field corresponding to two different workpiece speeds (*n*_w_ = 100 rpm and *n*_w_ = 200 rpm) when the grinding wheel linear speed *v*_s_ = 40 m·s^−1^ and grinding depth *a*_e_ = 0.9 μm. When the workpiece speed is increased from 100 rpm to 200 rpm, the maximum temperature increases from 145.1 °C to 165.3 °C. The increase in workpiece speed leads to an increase in the maximum undeformed cutting thickness, grinding force, and heat flux density in the grinding arc zone, ultimately leading to an increase in the temperature of the grinding arc zone. Compared with Figure 13, the influence of workpiece speed on the temperature field is relatively small because as the workpiece speed increases, the surface material of the raceway undergoes grinding and the processing time becomes shorter. After grinding, it is immediately cooled by the grinding fluid, so the maximum temperature growth rate is slower.

**Figure 15:**
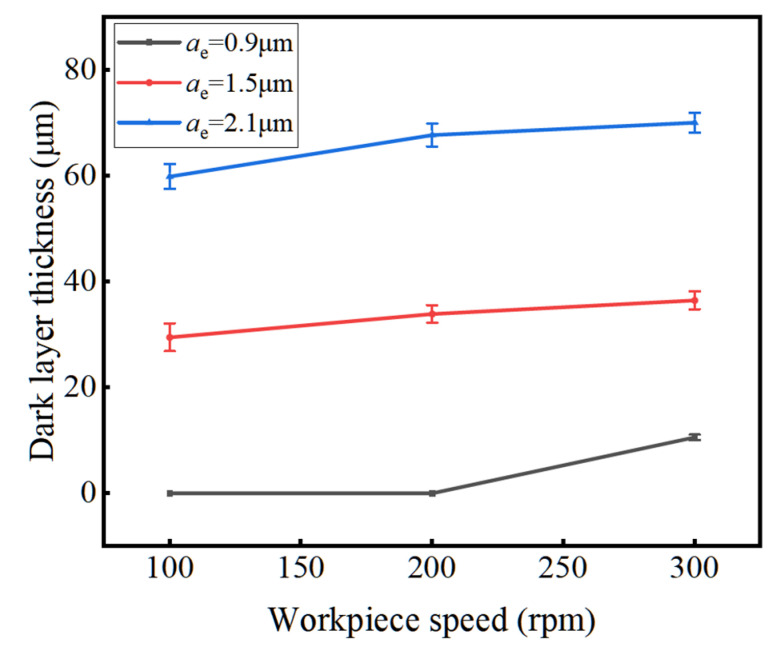
The influence of workpiece speed on the dark layer thickness.

Figure 15 shows the influence of workpiece speed on the dark layer thickness. As the rotational speed of the workpiece increases, the dark layer thickness slowly increases, corresponding to the slow increase in the temperature field in Figure 14. Similarly, the increase in workpiece speed leads to an increase in the maximum undeformed cutting thickness, resulting in an increase in grinding heat and the temperature at the grinding arc zone. However, due to the increase in workpiece speed, the grinding time of the surface material of the raceway becomes shorter, reducing the time for heat accumulation. After grinding, it is immediately cooled by the grinding fluid, so the dark layer thickness increases slowly.

## 6. Conclusions

In this paper, the grinding temperature field of the outer ring of the inner raceway of the tapered roller bearing was simulated using a finite element method and the grinding-affected layer thickness was deduced. Finally, the validity of the finite element model was verified using experimental results. The finite element model can predict the dark layer thickness in the grinding of the outer-ring inner raceway of the tapered roller bearing quickly and accurately, providing a rapid and low-cost improvement direction and exploring the unknown possibility for the grinding. And the finite element model also provides ideas and solutions for the grinding simulation of other type parts. The specific conclusions are as follows:

(1) Along the bearing outer ring raceway width direction, the thicker the bearing raceway, the lower the temperature. And the thinner the bearing raceway, the higher the temperature. The highest temperature was at the thin end surface near the center of the grinding arc.
(2) The experimental and simulated dark layer thickness values were compared, and the error was 9.8%, which verified the validity of the finite element model.
(3) The increase in grinding depth and workpiece speed will lead to the increase in grinding temperature. The influence of workpiece speed on grinding temperature was moderate, and the influence of grinding depth on grinding temperature was greater.
(4) The thickness of the dark layer increased with the increase in workpiece speed and grinding depth, and the influence of grinding depth was significant.

In this paper, the research results provided a theoretical basis for improving the grinding surface quality of the outer-ring inner raceway of the tapered roller bearings and eliminating the grinding-affected layer, and it had important guiding significance for optimizing process parameters and improving grinding efficiency.

## Figures and Tables

**Table 1:** Integral interval for various types of abrasives.

*h*_cu,max_	Type of Abrasives	*x*_min_	*x*_max_	*y*_min_	*y*_max_	*l*_min_	*l*_max_	Region
$h_{\mathrm{cu},\max}<\frac{d_{\max}\xi_{\mathrm{cut}}}{l_{i}/l_{c}}$	Plow	−*δ*/2	*x*_p_	*f*(*x*)	*g*(*x*)	*l*_plow_	*l*_c_	*II*
		*x*_p_	*δ*/2	*f*(*x*)	*d*_max_	*l*_plow_	*l*_c_	
		−*δ*/2	*x*_p_	*g*(*x*)	*d*_max_	*l*_plow_	*l*_cut_	*III*
	Cut	−*δ*/2	*x*_p_	*g*(*x*)	*d*_max_	*l*_cut_	*l*_c_	*III*
$h_{\mathrm{cu},\max}>\frac{d_{\max}\xi_{\mathrm{cut}}}{l_{i}/l_{c}}$	Plow	−*δ*/2	*δ*/2	*f*(*x*)	*g*(*x*)	*l*_plow_	*l*_c_	*II*
		−*δ*/2	*δ*/2	*g*(*x*)	*d*_max_	*l*_plow_	*l*_cut_	*III*
	Cut	−*δ*/2	*δ*/2	*g*(*x*)	*d*_max_	*l*_cut_	*l*_c_	*III*

**Table 2:** Material properties of GCr15.

Properties	Value
Hardness *H* (MPa)	3800
Shear yield strength *τ*_s_ (Mpa)	700
Frictional coefficient *μ*_d_	0.3
Frictional coefficient (chip and abrasive) *μ*_g_	0.3
Thermal conductivity of GCr15 *k*_w_ (W/mK)	34.3
Density *ρ*_w_ (kg/m^3^)	7810
Specific heat *c*_w_ (J/kgK)	778
Thermal conductivity of abrasives *k*_g_ (W/mK)	35
Convective heat transfer coefficient of coolant *h*_f_ (W/m^2^K)	15,000

**Table 3:** Thermophysical properties of GCr15.

Properties	Value					
Temperature *T* (°C)	20	100	200	300	400	500
Thermal conductivity *k* (W/mK)	43.8	42.7	40.8	38.5	35.6	32.4
Density *ρ* (kg/m^3^)	7834	7809	7780	7745	7712	7674
Specific heat *C* (J/kgK)	466	517	540	568	614	670

**Table 4:** Experimental groups and process parameters.

Number	Workpiece Speed/rmp	Grinding Depth/μm
1	100	0.9
2	200	0.9
3	300	0.9
4	100	1.5
5	200	1.5
6	300	1.5
7	100	2.1
8	200	2.1
9	300	2.1

**Table 5:** Simulation value of dark layer thickness.

Number	Workpiece Speed/rmp	Grinding Depth/μm	Feed Speed/μm·s^−1^	Dark Layer Thickness/μm
1	100	0.9	3	0
2	200	0.9	6	4.9
3	300	0.9	9	11.3
4	100	1.5	5	30.6
5	200	1.5	10	37.3
6	300	1.5	15	41.9
7	100	2.1	7	63.5
8	200	2.1	14	69.8
9	300	2.1	21	73.2

## Data Availability

Data are contained within the article.
